# Inflammation signals airway smooth muscle cell proliferation in asthma pathogenesis

**DOI:** 10.1186/2049-6958-8-11

**Published:** 2013-02-06

**Authors:** Mohammad Afzal Khan

**Affiliations:** 1 Department of Medicine, Stanford University, VAPAHCS, 3801 Miranda Avenue, Building 101, Room B4-105, Palo Alto, California, 94304, USA

**Keywords:** Airway inflammation, Airway smooth muscle cells, Asthma

## Abstract

**Background:**

Airway inflammation stimulates proliferation of airway smooth muscle cell, which contributes to the development of hyperplasia and hypertrophy of smooth muscle cell. The increase in airway smooth muscle cell mass is believed to be due to an up-regulation of inflammatory mediators in the airway. It is now well recognized that chronic inflammation as well as airway hyper-responsiveness and remodeling of airway during inflammation, are crucial to asthma. Airway hyper-responsiveness is caused by increased cell proliferation or by hypertrophy of airway smooth muscle cell depending on the nature of the inflammatory stimulation. Airway smooth muscle cell proliferation in asthma is regulated by the proinflammatory cytokines including IL-1β and TNF-α. These proinflammatory cytokines have been shown to influence human airway smooth muscle cell proliferation *in vitro,* which is due to cyclooxygenase-2 expression, production of prostaglandin E2, and increased cAMP levels.

**Conclusions:**

This review highlights the role of different proinflammatory cytokines in regulating airway smooth muscle cell growth and also focuses on regulation of differential gene expression in airway smooth muscle cell by growth factors and cytokines, also to bestow unique insight into the effects of conventional asthma therapies on airway smooth muscle cell proliferation and development of new therapeutic strategies to control asthma.

## Review

### Background

Asthma is a chronic, inflammatory disease of the lung characterized by intermittent airway obstruction, airway hyper-responsiveness, presence of activated inflammatory cells, inflammatory mediators, and remodeling in the airway [1-3]. Clinically, asthmatic patients show shortness of breath, wheezing, coughing, and chest tightness and decreased forced expiratory volume in one second, which is reversible with β-adrenergic receptor agonist administration. The role of inflammation in the pathogenesis of asthma has been reported by Tulic *et al*. [4] and the presence of inflammatory cell infiltration within the asthmatic airway suggests the contribution of the inflammatory process to asthma pathogenesis [4,5]. Airway hyper-responsiveness is results in enhanced airway narrowing in asthma patients [6]. Inflamed airway secretes histamine, thrombin, endothelin, tachykinins, and leukotrienes to mediate airway contraction [5,7,8]. It has been reported that inflammatory cytokines IL-1β and TNF-α increase the expression of bradykinin, tachykinins and leukotrienes receptors and Gαq and Gαi proteins, which can increase the contractility of airway smooth muscle cell (ASM) [9-12]. The increased in Gi protein-coupled receptor signaling has also been shown to activate Rho guanine nucleotide exchange factors and Rho, which can lead to contractile or calcium “sensitization”. Increased expression of myosin light chain kinase, RhoA and Gα12/Gα13 has been reported in allergic inflammation model of animal as well as in clinical patients [13]. Additionally, Glycoprotein YKL-40 remains elevated in asthmatic patients [14] and it leads to a significant increase in IL-8 production, which is dependent on MAPK (JNK and ERK), and NF-kB pathways activation [15,16].

### Airway inflammation

Inflammation is defined by infiltration of the airway by inflammatory cells including eosinophils, mast cell, monocytes, lymphocytes, active complement fragments (C3a and C5a), and neutrophils, which contribute to elevated levels of inflammatory mediators and procontractile stimulants [17-21]. C3a activates mast cells, basophils, eosinophils, and contraction of ASM [22]. It has been reported that C3a plays a crucial role in asthma primarily by regulating mast cell-ASM cell interaction. In clinical conditions of asthma, both infections and allergens of respiratory tract cause a local complement activation. The complement factors C3a and C5a play a central role in asthma pathogenesis because of their ability to recruit and activate leukocytes, increase vascular permeability, stimulate contraction of smooth muscle, and trigger degranulation of mast cells. It has been reported that bronchoalveolar lavage of asthma individuals contains quantitative higher levels of both C3a and C5a as compared with healthy controls. It has also been demonstrated that ASM cells modulate airway inflammation by release of cytokines and chemokines and they express receptors and adhesion molecules that modulate inflammatory cell trafficking and function. When exposed to IL-1β, TNF-α, IFN-γ, IL-4, IL-13, and bradykinin inflammatory mediators in-vitro, ASM cells have been shown to release IL-8, eotaxin, monocyte chemoattractant proteins (MCP)-1, -2, and −3, GM-CSF, IL-5, regulated upon activation, normal T-cell expressed and secreted (RANTES, CCL-5), IL-6 and other IL-6 family cytokines including leukemia inhibitory factor (LIF) and IL-11 [23,24]. In addition to this, few studies reported expression of RANTES and eotaxin in ASM cells in bronchial biopsies from asthmatic patients, and suggested that ASM cells also exhibit these synthetic functions *in-vivo*[25]. These cytokines specifically modulate migration and survival of eosinophils, mast cells, monocytes, and lymphocytes, as well as immunoglobin production. ASM also expresses intracellular adhesion molecule-1 and vascular cell adhesion molecule-1 (VCAM1), CD44, as well as integrins, which allow interaction with T cells and enhance T cell survival or modulate T cell inflammatory functions [26,27].

### Airway remodeling

**Figure 1:**
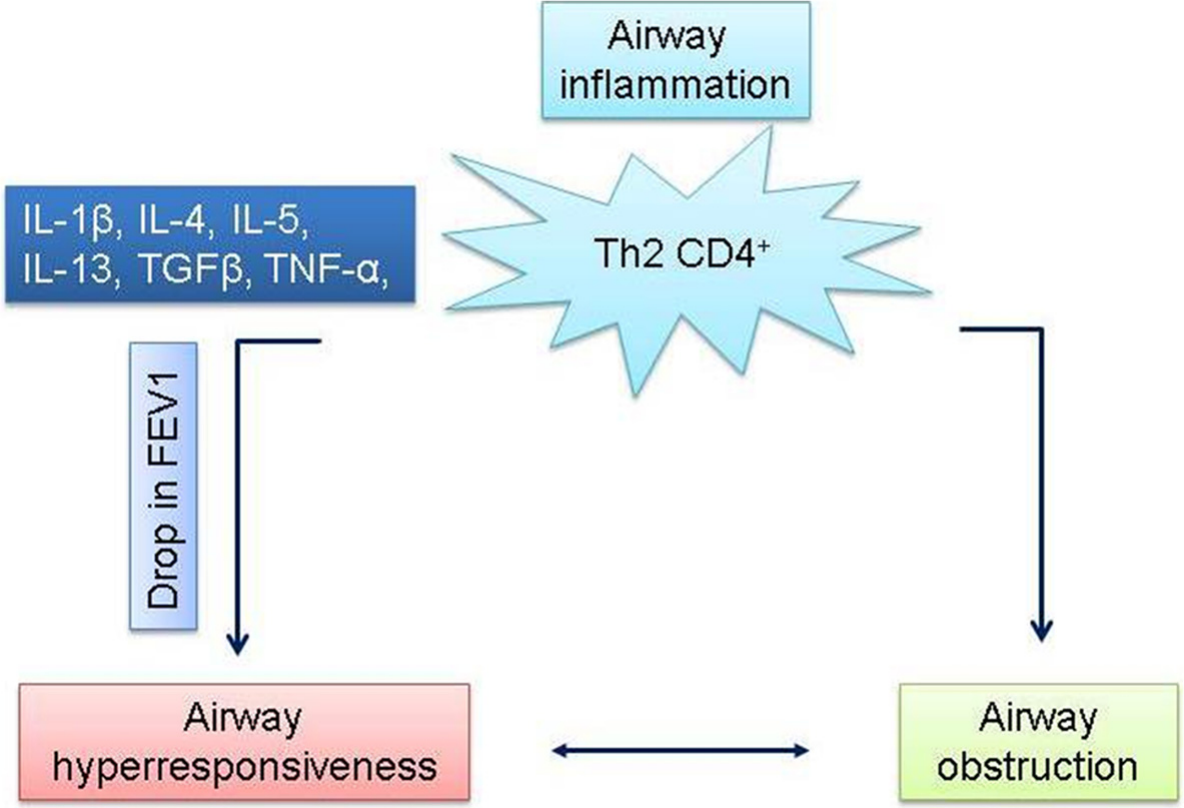
Model illustrates how Th2 driven cytokines induce airway hyper-responsiveness and obstruction in asthma condition.

It has been reported from human autopsy and biopsy studies that the degree of airway remodeling is associated with abundance of inflammatory cells and mediators in the airway [6,17,19]. In addition, few researches have shown increased vascularity of the airway, which may contribute to thickening of the airway wall. However, in both human asthmatics and animal models of asthma, increased ASM mass has been reported but mechanisms of this pathological condition are still not well clear. A number of researches have suggested that TGF-β appears to induce ASM hypertrophy [25]. In spite of the relative contribution of hyperplasia or hypertrophy, all asthma conditions show altered airway mechanics and increased airway resistance. The actual underlying relationship between inflammation and airway remodeling is due to either chronic inflammation as seen in animal model of asthma and in airway eosinophilia in wheezing of young children, suggesting that remodeling may be an early pathogenic mechanism [6,17,19,25,28,29]. In addition to the functional changes associated with ASM, the structural changes are also playing a crucial role in promoting excessive airway narrowing as seen in elastolytic mouse model of emphysema [30,31]. Increase in airway wall thickness, ECM remodeling, and detachment from the lung parenchyma and elastic recoil of the lung are supposed to alter the tension on ASM allowing for enhanced shortening and greater degree of airway narrowing [32]. In spite of this, the major effect of airway remodeling is due to an increase in ASM mass and the changes associated with airway geometry during airway contraction [32,33] (Figure 1). 

### Mechanisms of ASM proliferation

Initial studies of the role of ASM in asthma focused on examining contractile properties of ASM cells isolated from animal or human trachea. Eosinophils have been hallmark of asthma inflammation and actively contribute in ASM cell proliferation and cell mass [34]. *In-vitro* culture has shown that ASM preserves functional responses to specific stimulant including bradykinin, thromboxane A2, histamine, leukotriene D4, platelet derived growth factor α, or β-agonists, as well as expresses ion channels [1]. Epidermal growth factor, platelet derived growth factor and basic fibroblast growth factor activate receptor tyrosine kinase and have shown potent ASM mitogenic properties *in vitro*. In ASM cells, subsequent activation of receptor tyrosine kinase, phosphoinositide-3 kinase and p42/p44 extracellular signal-regulated kinases results in initiation of ASM proliferation. G protein couples receptors have also been shown to stimulate ASM proliferation and their levels are found elevated in the asthmatic airway. Additionally, GPCR ligands have been reported to up regulate growth factor-stimulated growth of human ASM and co-stimulation of ASM cells with epidermal growth factor and thrombin, histamine or carbachol induce ASM cell proliferation. These stimulatory signals were found to increase GPCR mediated activation of phosphoinositide-3 kinase. Other mediators including the cytokines, chemokines and cytokine receptors also play a crucial role in asthma pathogenesis and development of ASM proliferation. Chemokines mainly recruit immune cells to the site of inflammation. Chemokine receptors have been classified according to their function, and CCR3 is the most relevant receptor and it controls eosinophil recruitment by eotaxin and is also expressed on lymphocytes. Newly tested antisense oligonucleotides bind (TPI ASM-8) to complimentary mRNA of chemokine receptors CCR3 [35], thereby suppressing gene transcription. In most of research findings in asthma models and clinical samples, it has been reported that Th2 cytokines IL-4, IL-5 and IL-13 or TGF-β and IL-6 play a crucial role due to their possible role in airway remodeling. The treatment of ASM with IL-1α and TNF-α attenuated the mitogenic effects of bFGF and thrombin, but strongly increased mitogen-stimulated growth in presence of indomethacin or dexamethasone, which was associated with suppression of COX-2 expression and PGE2 production. A substantial documentary evidence supports IL-1β and TNF-α as a central players in the pathogenesis and progression of asthma; they are also common in any inflammatory disorder, and can act both locally and systemically. Elevated levels of IL-1β and TNF-α are reported from BAL fluid of asthma patients and they increase with severity of disease. IL-1β and TNF-α have been shown to act on airway inflammatory cells, and modulate effects of other cytokines and ASM cells. IL-1β also functions in cooperation with other cytokines such as IL-5 or GM-CSF, promotes eosinophil survival, and modulates ASM function. It has been reported that stimulation of ASM cells with IL-1β or IL-1β and TNF-α leads to sensitization of adenylatecyclase and elevated cAMP production in response to Gs protein-coupled receptor stimulation. The regulatory effects of IL-1β and TNF-α on ASM cell proliferation could have important consequences for development of asthma therapeutics. Drugs such as (COX-2-targeting) non-steroidal anti-inflammatory drugs and glucocorticosteroids are often used to treat inflammation-based diseases but their use is associated with significant side effects. The ability of glucocorticosteroids to suppress ASM COX-2 and PGE2 induction caused by inflammatory agents such as IL-1β and TNF-α could represent a deleterious effect of glucocorticosteroids treatment.

## Conclusions

Airway diseases are characterized by changes in composition of the airway wall; in fact, these changes are believed to be largely responsible for the various features of those diseases. For example, asthma is characterized by wall thickening (including both increased ASM and connective tissue) and ASM hyper-responsiveness. Inflammation causes airway hyper-responsiveness by up-regulation of procontractile agonists in the airway, increased expression of receptors, their signaling intermediates, and effectors, as well as regulators of calcium stores in ASM.

**Figure 2:**
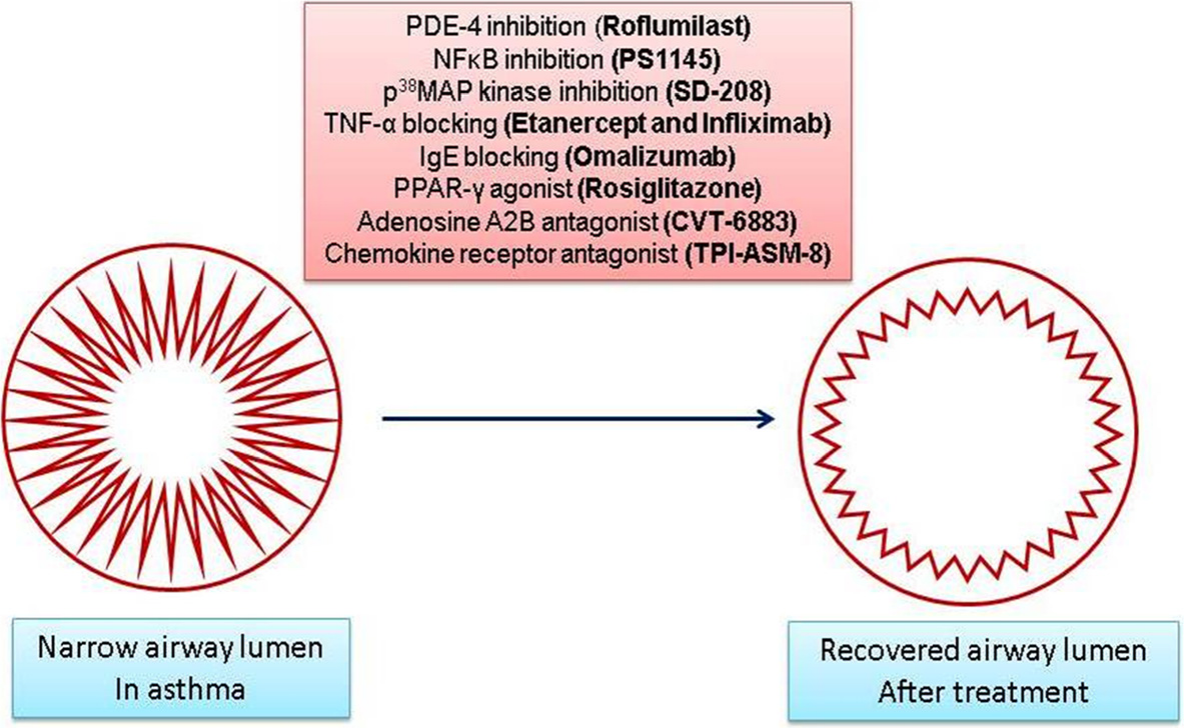
Model illustrates details of asthma treatment strategies.

Studies of the airways in health and disease often use indices of the degree of ASM contraction: *e.g.*, measures of airflow resistance in patients (forced expiratory volume in 1 second, FEV_1_) or animal models, airway narrowing in lung slices, or grams tension in isolated ASM strips [30,31]. Increased ASM mass, seen in asthma and various animal models of asthma, is expected to manifest as changes not only in the magnitudes of airway narrowing, but also in the rates of narrowing [30,31]. Thus in asthma, ASM appears to cooperate in most known pathogenic processes. ASM is central to the development of Airway Hyper-Reactivity (AHR) being the target and effector of procontractile agonists. Increases in ASM mass affecting airway geometry and mechanics, in conjunction with contractile sensitization, have profound effects on airway lumen size and resistance to airflow. Through both direct and indirect effects on other resident and infiltrating airway cells, ASM plays an important role in modulating the inflammatory response, and thereby promotes many of the features of airway remodeling. Likely asthma is caused by interactions between genetic and environmental agents, but its pathogenesis is still not well known. Clinically, asthma patients are reported to have elevated numbers of CD4^+^ T (Th2 subtype) cells in the airways. These Th2 lymphocytes produce IL-4, IL-5, IL-9, and IL-13 which mediate the inflammatory response in asthmatic airways by recruiting and activating additional Th2 cells and also mast cells and eosinophils. Serine and matrix metalloproteinase have been reported as one of the crucial agents in asthma pathogenesis. A number of new therapeutic targets to rescue patients from asthma are underway. Omalizumab is a recombinant humanized monoclonal antibody that is proven to be effective for patients with moderate-to-severe persistent asthma [36,37]. Omalizumab binds to IgE and produces anti-inflammatory effects in allergen challenge and bronchial biopsy protocols supported the role that IgE plays in airway inflammation in asthma [38]. TNF-α also is up-regulated in asthma and promotes recruitment of neutrophils and eosinophils into the airways [5,29,34]. Etanercept a recombinant fusion protein that blocks TNF-α, produces marked and significant improvement in asthma control when added to high-dose ICS therapy in patients with treatment-resistant asthma [39,40]. The anti-TNF-α monoclonal antibody infliximab shows positive outcomes in moderate asthma despite ICS therapy [41]. Other therapies include selective inhibition of Cyclic adenosine monophosphate (cAMP), Macrolides and ketolides antibiotics, Adenosine A2B antagonists [42,43], Chemokine/chemokine receptor antagonists [44,45], prostanoid and F2-isoprostane antagonists, peroxisome proliferator-activated receptor gamma agonists, nitric oxide donors and inducible nitric oxide synthase inhibitors, and toll-like receptor modulators (Figure 2). A novel antisense oligonucleotide, TPI ASM-8, has been targeted against CCR3 [35] and other chemokine receptors β-chain of the proinflammatory cytokines IL-3, IL-5 and GM-CSF, which are involved in Th2 inflammation. TPI ASM-8 is an inhaled therapy allowing specific targeting to the lungs of a therapy that acts by different mechanisms to inhibit the activity of eosinophils and other Th2 cells, such as mast cells and basophils. These agents modulate inflammatory cell function and/or reduce airway inflammation in experimental models [39,46-48]. The last few years have seen a concerted effort by the National Institutes of Health and other agencies to manage asthma. The therapeutic and attitudinal advances in managing asthma have been very substantial in the past 15–20 years, resulting in more effective and safer ways of controlling it. 

In summary, development of new research findings of asthma pathophysiology will help in designing novel therapeutic targets for asthma cure.

## Abbreviations

AHR: Airway Hyper-Reactivity; ASM: Airway smooth muscle; BAL: Bronchoalveolar lavage; BFGF: Basic fibroblast growth factor; C3a/C5a: Complement anaphylatoxins; COX-2: Cyclooxygenase; CAMP: Cyclic adenosine monophosphate; ECM: Extracellular Matrix; GPCR: G protein coupled receptors; LIF: Leukemia inhibitory factor; MAPK: Mitogen-activated protein kinases; MCP: Monocyte chemoattractant proteins; PGE2: Prostaglandin E2; VCAM1: Vascular cell adhesion molecule-1.

## Competing interests

The author declares that he has no competing interests.
